# Novelty of harnessing electromagnetic fields to boost graphene oxide nano particles antibacterial potency

**DOI:** 10.1038/s41598-025-91408-y

**Published:** 2025-03-19

**Authors:** Mai I. El-kaliuoby, Ashraf Morsy, Ahmed H. Abdel-Salam, Ahmed Morsy, Ahmed M. El‑Khatib, Alaa M. Khalil

**Affiliations:** 1 Physics and Chemistry Department, Faculty of Education, Alexandria University, Alexandria, 21544 Egypt; 2 Faculty of Engineering, Petrochemical Department, Pharos University in Alexandria, Canal El Mahmoudia Street, Beside Green Plaza Complex 21648, Alexandria, Egypt; 3 Institute of Graduate Studies and Research (IGSR), Alexandria University, 163 Horreya Avenue, El-Shatby, Alexandria, Egypt; 4 Chemistry Department, Faculty of Science, Alexandria University, Alexandria, Egypt; 5 Chemistry Department, Faculty of Science, University of Jeddah, Jeddah, Saudi Arabia; 6 Faculty of Dentistry and Oral Surgery, Pharos University in Alexandria, Canal El Mahmoudia Street, Beside Green Plaza Complex 21648, Alexandria, Egypt; 7 Physics Department, Faculty of Science, Alexandria University, Alexandria, 21511 Egypt; 8 Basic Sciences Department, Faculty of Engineering, Pharos University in Alexandria, Canal El Mahmoudia Street, Beside Green Plaza Complex 21648, Alexandria, Egypt

**Keywords:** Graphene oxide nanoparticles (GONPs), Electromagnetic waves (EMWs), Antibacterial efficacy, Reactive oxygen species (ROS), Pseudomonas aeruginosa, Biochemistry, Biophysics, Chemistry, Engineering, Mathematics and computing

## Abstract

The urge need for innovative integration between Electromagnetic Waves (EMWs) and nanotechnology offers exciting possibilities for improving antimicrobial treatments to combat antibacterial resistant bacterial infections. This study explores how EMWs at range below 300 Hz can enhance the antibacterial efficacy of Graphene Oxide Nanoparticles (GONPs) against *Pseudomonas aeruginosa*, a significant pathogen in antibiotic resistance. EMWs at range below 300 Hz, interact with bacterial cell membranes to affect ion channels, permeability, and cellular signaling, offering a non-invasive method to amplify antimicrobial effects. GONPs synthesized through glucose pyrolysis and characterized by X-ray diffraction, UV-visible spectroscopy, high-resolution transmission electron microscopy, and Fourier-transform infrared spectroscopy, exhibit potent antibacterial properties due to their sharp edges, large surface area, and ability to generate Reactive Oxygen Species (ROS). These nanoparticles disrupt bacterial membranes, form biofilms, and damage cellular components through oxidative stress. The study examines how those EMWs can enhance GONP penetration into bacterial cells, increase ROS production, and disrupt biofilms. By optimizing EMWs parameters such as frequency, intensity, and duration this research aims to develop new, non-invasive antibacterial therapies. The results could lead to advanced antimicrobial strategies, integrating nanotechnology with electromagnetic field exposure, offering innovative solutions to address antibiotic-resistant infections and improve treatment efficacy. This approach represents a significant step toward more effective, targeted antibacterial therapies.

## Introduction

The increasing prevalence of antibiotic resistance and the limitations of traditional antimicrobial therapies have highlighted the urgent need for innovative strategies to combat bacterial infections effectively^1,2^. This has led to significant interest in exploring synergistic approaches that combine antimicrobial agents with external physical stimuli such as electromagnetic waves (EMWs). Among these, EMWs at range below 300 Hz, has shown particular promise^3,4^. EMWs interact with bacterial cell membranes through mechanisms such as ion channel modulation, alterations in membrane permeability, and cellular signaling pathways, resulting in various biological effects^5,6^. Their ability to penetrate biological tissues with minimal absorption makes them well-suited for non-invasive antibacterial therapies^7^. Recent research has demonstrated that EMWs at range below 300 Hz can inhibit bacterial growth, disrupt biofilms, enhance antibiotic uptake, and amplify the bactericidal effects of antimicrobial agents^8,9^. This has led to investigations into combining those EMWs with nanoparticles (NPs) to enhance antimicrobial efficacy against multidrug-resistant bacterial strains^10,11^. Studies suggest that EMWs at that frequency range can facilitate NP penetration into bacterial cells, increase reactive oxygen species (ROS) production, and disrupt cellular metabolism, thereby enhancing the antimicrobial activity of NPs^12,13^.

Graphene oxide Nano particals (GONPs) have emerged as highly effective antibacterial nanomaterials due to their unique properties, including sharp edges, large surface area, and high stability^14^. These attributes enable GONPs) to disrupt bacterial cell membrane integrity, leading to leakage of cellular contents and cell death^15,16^. GONPs also interfere with biofilm formation and promote the dispersion of existing biofilms, which is crucial for combating biofilm-associated infections^17,18^. Their ability to generate ROS contributes to oxidative stress within bacterial cells, damaging critical cellular components such as DNA, proteins, and lipids^19,20^. The conductive properties of GONPS, due to their π-conjugated electron systems, suggest that they might interact synergistically with those EMWs^21,22^. GONPS’ functional groups, which can ionize in aqueous solutions, form charged species that may interact electrostatically with bacterial cell surfaces, enhancing their antibacterial effects^23,24^. Additionally, the localized electric fields generated by GONPS can exert forces on the bio-charges of cell membrane macromolecules, potentially increasing the susceptibility of bacteria to antimicrobial agents^25,26^. This study aims to explore how EMWs at range below 300 Hz can enhance the antibacterial efficacy of GONPS against *Pseudomonas aeruginosa* (*P. aeruginosa*), a common and clinically significant pathogen. We seek to optimize the exposure parameters of those EMWs, such as frequency, intensity, and duration, to assess their impact on bacterial cell viability, biofilm disruption, and Reactive Oxygen Species (ROS) generation^27,28^. By investigating these interactions, our research could contribute to new non-invasive therapies^29–31^ methods for addressing bacterial infections, particularly those resistant to conventional antibiotics^32–34^. The insights gained could lead to the development of advanced antimicrobial materials and devices that integrate nanotechnology with electromagnetic field exposure^35^. Ultimately, this work aims to foster a shift towards more effective and targeted antibacterial treatments, offering new solutions in the fight against antibiotic resistance and persistent bacterial pathogens.

## Materials and methods

### Materials

The glucose (MW 180.15) was sourced from Adwic El Nasr Pharm. Chem. Co. We procured a 25% ammonia solution from Solute Chem in Germany. Methanol (purity > 99%) was obtained from Labsolve, Lisbon, Portugal.

### Methods

#### Synthesis of GONPs

GONPs were synthesized through the pyrolysis of glucose. The glucose was heated to 250 °C using a hot plate, resulting in a liquid state within five minutes. Over the next 20 min, the liquid color changed from colorless to yellow and then to orange. To form the GONPs, 100 ml of a 12.5% ammonia solution was gradually added to the orange liquid while stirring vigorously. The mixture was then heated at 70 °C for three hours until the ammonia odor dissipated and the pH reached 7.0. The GONPs were characterized using several techniques. UV-visible spectra were acquired with a Thermo USA (Evolution 600) spectrophotometer. X-ray diffraction patterns were recorded with a Shimadzu-Japan (7000) diffractometer. The high-resolution transmission electron microscope (HRTEM) [JEOL (JEM-2100 LaB6)] was used to determine the particle size and crystal structure. Fourier-transform infrared (FTIR) spectroscopy was employed to analyze the chemical structure of the membranes, using the Spectrum BX 11 Infrared Spectrometer FTIR LX 18-5255 from Perkin Elmer.

### Electric and magnetic fields’ exposures

It is worthy to state that, the exposure protocols are maintained within the safety limits defined typically by International Commission on Non-Ionizing Radiation Protection (ICNIRP) and the World Health Organization (WHO)^36^. Our current research builds upon the previous findings on integration between nanotechnology and exposure to EMWs, and we are continuing to explore this area on the basis of optimum exposure former results such (frequency range, field intensity and exposure periods)^37,38^.

### Electric field exposure system

**Fig. 1:**
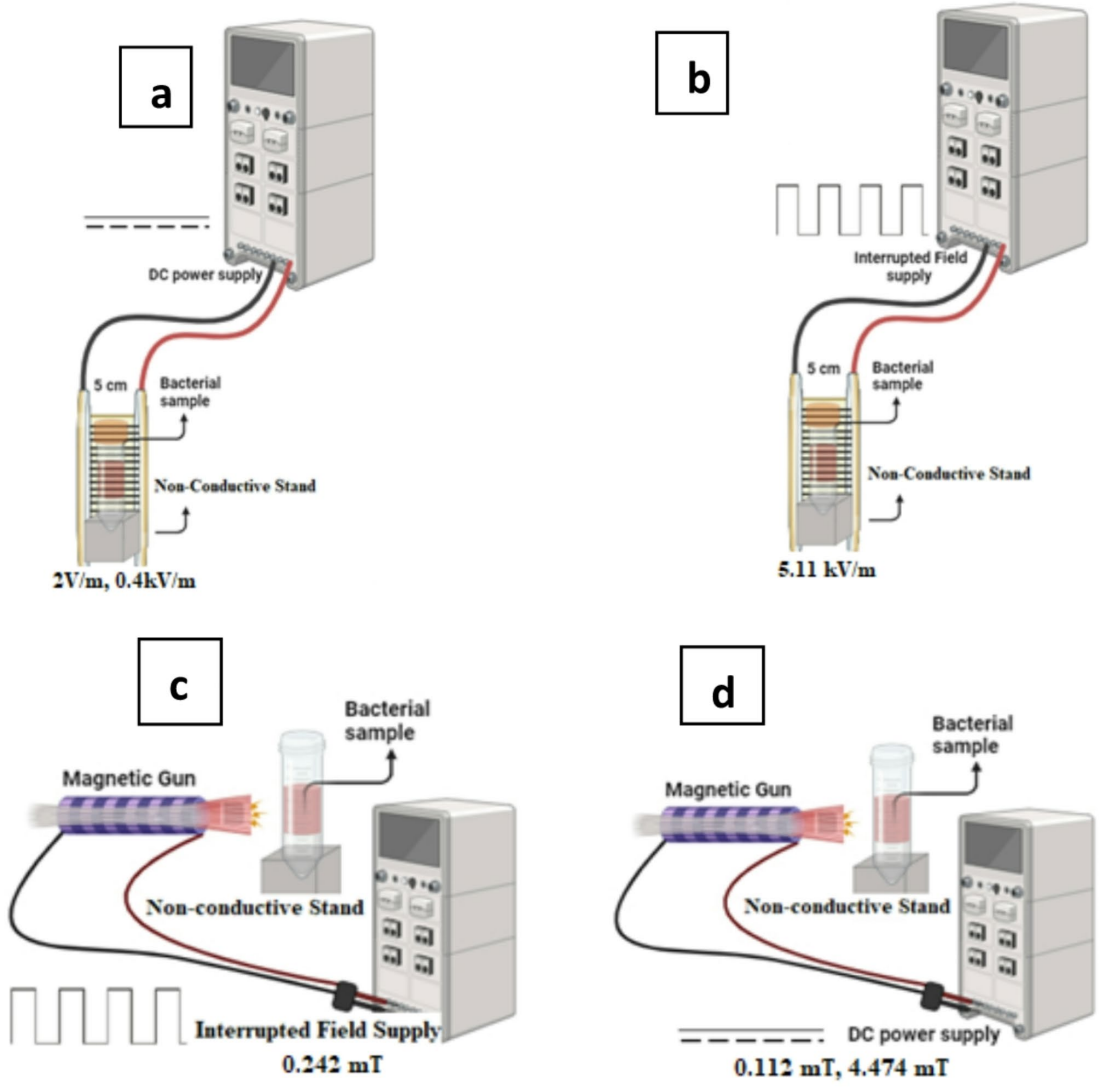
Schematic diagram for the electric fields exposure systems. (**a**) DC electric field at 2 V/m and 0.4 kV/m; (**b**) pulsed electric field of 5.1 kV/m. The magnetic fields exposure systems; (**c**) static magnetic field at 0.112 mT and 4.474 mT; (**d**) pulsed magnetic field of 0.242 mT.

A system of two parallel conductors connected to a power supply is maintained to supply a capacitive electric field between the two conductors. Two exposure protocols are performed for tested samples. One protocol is maintained by using DC power supply (Yaadgoing YD 305D) to deliver 100 mV and 20 V over the connected electrodes (5 cm apart) to achieve two electric fields of 2 V/m and 0.4 kV/m within field area between the electrodes. For the other exposure protocol a DC power supply is locally manufactured (Physics Lab of the Alexandria University Faculty of Science, Alexandria, Egypt) to convert input DC voltage of 6 V into amplified interrupted voltage up to 400 V through an electronic switching device of 50% duty cycle and DC/DC voltage converter and exposure is performed at two frequencies of 0.7 Hz^36^ and 50 Hz. The samples under exposure test are placed midway between the two capacitor circular shape parallel conductors of diameter 4 cm. The supernatant glass tubes containing bacterial samples are placed on top of a nonconductive stand and the temperature is adjusted during the treatment period to be 27 °C by using an electronic thermometer (SP Bel-Art, H-B DURAC Calibrated Electronic Thermometer, China). The distance between the parallel conductors is adjusted to be 5 cm and interrupted voltage of 250 V is applied to maintain homogenous electric field of 5.11 ± 0.13 kV/m as measured by field meter (Tri-field TF2 EMF meter (USA) of accuracy of ± 5%) at the point at which supernatant samples are placed. The applied electric field is generated in square pulsed form as displayed by oscilloscope type GOS-620 (GW Instek). For all experimental exposure work the time is adjusted to be 30 min and interrupted instantaneously every while just to shake the supernatant samples for ensuring the homogeneity of bacteria distribution in the broth media under exposure treatment^38^. For the safety limits it is worth to state that the exposure is within the safe limits that have been addressed by the International Commission on Non-Ionizing Radiation Protection (see: www.icnirp.org). For presentation a schematic diagram for the exposure systems are shown in Fig. 1a–b.

####  Magnetic field exposure system

A magnetic field exposure system is maintained for tested samples by two protocols, on by using DC power supply and the other by using interrupted field supply. The first protocol is performed by using DC power supply (Yaadgoing YD 305D) at two conditions 200 mV, 30 mA and 7.6 V, 1.2 A. The second protocol is performed by pulsed magnetic field system to apply interrupted field by using a magnetic gun of 445 turns coil and total resistance of 6.8 ohms. The magnetic gun of core length 0.15 m is connected to power supply that drive 65 mA current interrupted frequently at 50% duty cycle through an electronic switching device. Similarly, as formerly mentioned, the supernatant glass tubes containing bacterial samples are placed on top of a nonconductive stand and the temperature is adjusted during the treatment period to be 27 °C by using an electronic thermometer type SP Bel-Art, H-B DURAC Calibrated Electronic Thermometer (China). The samples under exposure protocols are placed at 0.005 m in front of the magnetic gun and its field intensity is measured at that point by using Gauss/Tesla meter model 4048 with probe T-4048.001 (USA) of accuracy ± 2%^39–41^. The fields under conditions of the first protocol are measured to be (0.112 ± 0.006) mT, and (4.474 ± 0.033) mT and found to be (0.242 ± 0.012) mT for the second protocol condition. The square pulsed magnetic field shape is shown on oscilloscope by using Linear Hall-effect IC sensor and displayed to be square unipolar pulses. It is in worth to state that, because of the very weak intensity of the applied magnetic fields its application is challenging as it could be interrupted by external other nearby fields and therefore the system is isolated to make sure that the generated field isn’t interrupted by any other external nearby electromagnetic fields. For presentation a schematic diagram for the exposure systems are shown in Fig. 1c–d.

### Antimicrobial activity

*P. aeruginosa* standard bacterial strain type ATCC 27,853 is used to be treated by GONPs alone and combined with exposure to electric and magnetic fields. The antibacterial impact is studied by agar well diffusion method to quantify the amount of survival bacterial^42^. The inoculation adopting condition was starting with approximately 10^5^ colony-forming units (CFU) of bacterial concentration to be constant during the experimental process. To prepare a fresh bacterial strain a subcultures are maintained every while and incubated for 24 h at 37^o^C. An amount of 0.5 McFarland (1.5 × 10^8^ CFU/ml) of bacterial cells is cultured as initial concentration for further experimental treatments. The bacteriostatic and bactericidal potentials of GONPs against P. aeruginosa are determined by monitor the lowest concentration of GONPs at which no visible bacterial growth as MIC and the lowest concentration of GONPs at which all bacterial cells are dead as MBC^43^. For that a twofold serial dilutions of GONPs started from 5 µg/µl till 0.0012 µg/µl are prepared by using nutrient broth as diluent^44^. Standard amounts 0.5 McFarland of bacterial cells are supplied to the serial dilutions to specify the MIC and MBC values after incubation at 37 °C for 24 h^45^.

The viability of bacterial cells is obtained to reflect the influence of exposure conditions and calculated as percentages relative to samples treated by GONPs at chosen concentration less than MIC as follows: viability % = [(average count of positive control -average count of treated)/average count of positive control] x 100^46,47^. It is worthy to state that the samples without any treatment are considered as negative control samples whereas the samples treated with GONPs are considered as positive control ones and samples treated by GONPs combined with exposure to electric or magnetic fields are considered as treated samples. Further, the kinetics of bacterial growth are monitored by obtaining turbidity in terms of optical density (OD at 600 nm) for broth medias inoculated by control and treated bacterial samples. Initially, the nutrient broth is chosen as reference media for relative comparisons. Growth curves to show the kinetics of growing is graphed between the obtained OD and incubation time up to 18 h for all samples under treatment. Moreover, the dynamics of bacterial growth are determined by analyzing the growth curves and calculating their arbitrary rate constants as follows: Arbitrary growth rate constant = 1/t ln (N/N_0_), where N is the count of bacterial cell at time (t) and N_0_ the initial cell count. Furthermore, the arbitrary rate constants are trended and graphed versus GONPs concentrations (< MIC)^48^.

In addition, the bacteria cytotoxicity as affected by GONPs and exposure to electric and magnetic fields is assessed by measuring the lactate dehydrogenase (LDH), protein and nucleic acid leakage levels. Following method adopted by Kim et al.^49^, the LDH levels are measured as relative changes to the negative control samples using a micro-plate spectrophotometer system (Spectramax190-Molecular Devices) at 490 nm. On the other hand, the protein leakages relative to the negative control samples in the broth media are obtained by using the Coomassie Protein assay reagent (Pierce, Rockford) as adapted by Li et al.^50^ and measured by the Bradford method at 595 nm. Finally, the evaluation of nucleic acid leakage was obtained by measuring the amount of acid released at 260 nm as adopted by Reddy et al.^51^ and it was repeated for error minimization^52^. The data are represented as percentages of changes as follows: Parameter% = [(treated samples-control samples)/control samples] × 100.

### Grouping scheme

The bacterial samples are divided into control and exposed groups based on the treatment conditions. The control groups as mentioned early, untreated bacteria as the negative control and bacteria supplemented with GONPs as the positive control. The exposed bacteria sample groups, one is exposed to static fields, and the other is exposed to pulsed electric and magnetic fields.

### Statistical analysis

The obtained data are presented as average mean values with its calculated standard deviation. The differences between mean values are calculated for all results and significance of difference is determined by using one-way analysis of variance (ANOVA). “*p*” is defined as be statistically significant at “*p* < .05” and statistically highly significant at “*p* < .001as performed by using the post hoc Tukey test^53^. Windows statistical package program (SPSS Inc., ver. 21) is used for all statistical analysis.

## Results and discussion

### Structural analysis

**Fig. 2:**
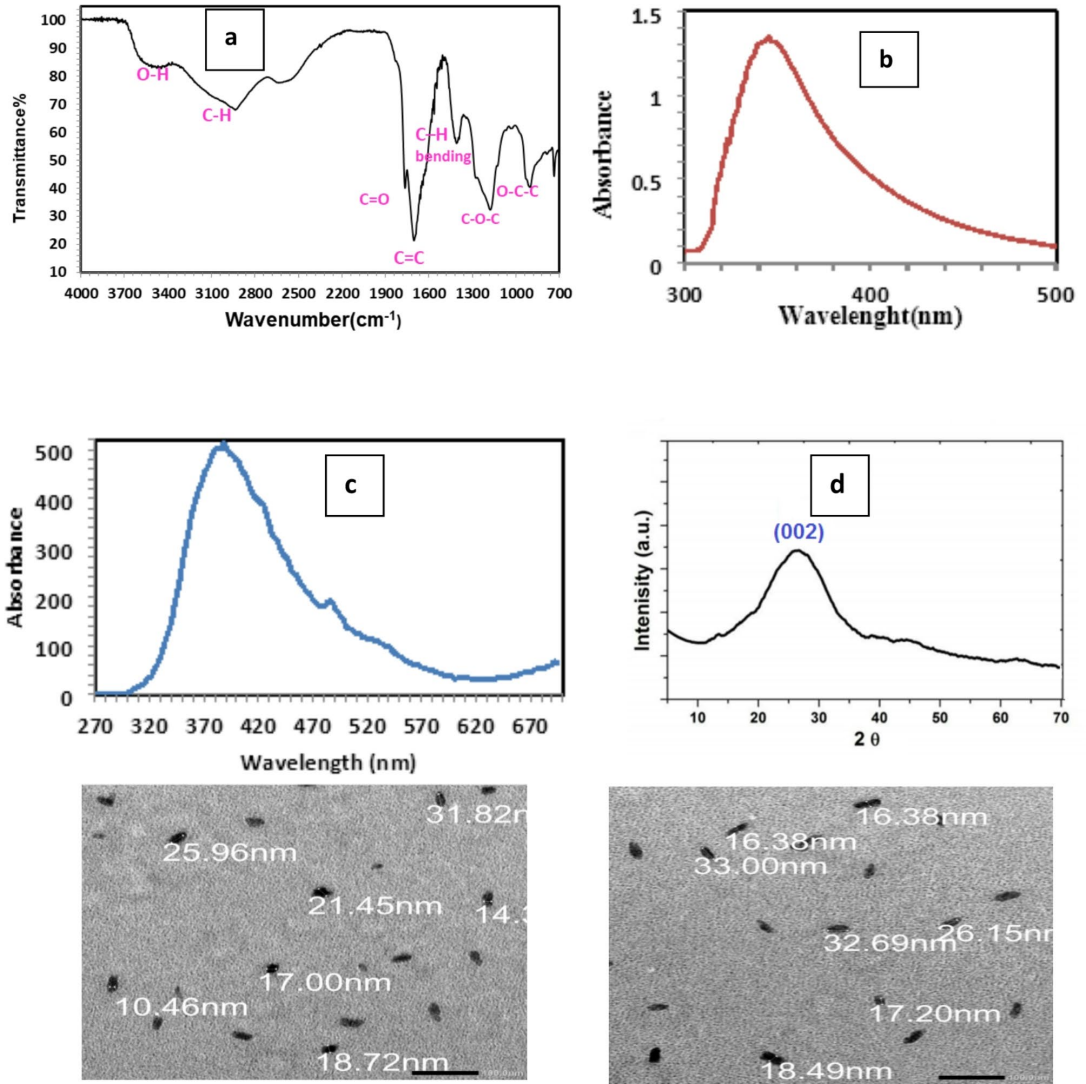
(**a**) Shows the FTIR spectra of the GONPS; (**b**) illustrates the UV-Vis spectra of GONP_S_ (**c**) displays the photoluminescence (PL) of GONPS under UV light; (**d**) XRD pattern of GONPs; (**e**) TEM images for nano graphene oxide.

The FTIR spectra of Nano Graphene Oxide (GONPs) reveal several distinctive bands related to various functional groups Fig. 2a. The peak at 1173 cm^−1^ corresponds to C-O-C stretching vibrations, characteristic of ether linkages in the GONPs. Bands at 898 cm^−1^ are associated with O-C-C groups, also indicative of ether linkages. A peak at 1698 cm⁻¹ represents C = C stretching vibrations, suggesting the presence of double bonds. The 1405 cm^−1^band is due to C-H bending, typical of alkanes, while the band at 1760 cm^−1^ corresponds to C = O ester groups, indicating ester bonds. Peaks at 2931 and 2621 cm⁻¹ are attributed to (C-H) stretching vibrations in alkanes. Broad OH bands at 3545 cm^−1^ signal the presence of hydroxyl groups^54^.

Optical properties were examined through photoluminescence (PL) and UV-Vis absorption spectra. The UV-Vis spectrum revealed a prominent absorption band centered at around 348 nm, related to the n-π* transition of C = O (Fig. 2b). Quantum dots, being semiconductor nanoparticles, show peak positions influenced more by the synthesis process than by size or shape^55^. PL spectra recorded from 240 to 420 nm excitation wavelengths exhibited variations in emission peaks, with a notable peak at 380 nm observed at a 350 nm excitation (Fig. 2c). This photoluminescence is attributed to the geminate recombination of electron-hole pairs in sp² clusters and is influenced by quantum confinement and edge effects^56^.

### X-Ray diffraction patterns

The X-ray diffraction (XRD) pattern of the GONPs is shown in Fig. 2d. It exhibits a broad (002) peak at 2θ = 26°, corresponding to an interlayer spacing of approximately 0.34 nm. This broad peak indicates that the GONPs have a smaller particle size compared to crystalline graphite. The lack of a distinct peak at 2θ = 19°, which is typically observed for graphene oxide, suggests that the processed sample consists of pure GQNPs, as similarly noted by Su et al. (2013)^57^.

### Transmission electron microscopy (TEM)

The Transmission Electron Microscope (TEM) data, presented in Fig. 2e, is utilized to assess various properties of nanoparticles, including particle size, sample or grain size, crystal structure, and atomic arrangement^58^. TEM provides detailed diffraction patterns from magnified images, revealing precise nanoparticle dimensions. The analysis of Nano graphene oxide particles via TEM indicates that they are within the nanoscale, with sizes observed to be below 35 nm. This confirms that the particles are indeed nanometer-sized^59^.

### Bacteriostatic and bactericidal GONPS potential

The potential of using GONPs is studied qualitatively and quantitatively. The MIC and MBC values are determined for *P. aeruginosa* samples treated with serial dilutions of GONPs (5.000 µg/ml: 0.0012 µg/ml) to represent the bacteriostatic and bactericidal effects of GONPS as tabulated in Table 1. The MIC data obtained showed *P. aeruginosa* with no visible bacterial growth after overnight incubation in broth media to be (> 0.039 µg/ml). On the other hand, MBC level appointed to be (> 0.078 µg/ml) when three or fewer colonies are observed (> 99.9% killed) in inoculated agar plates^60^. Samples of *P. aeruginosa* are inoculated at constant concentration of 1.5 × 10^8^ CFU/ml in broth media and exposed to EF and MF alone and supplement with 0.0024 µg/µl of GONP_S_ (lower than MIC).

**Table 1:** List of *P. aeruginosa* counts (CFU/ml ± SE) under supplement of GONPS at different concentrations.

Conc. of GONPS (µg/ml)	Count of *P. aeruginosa*(CFU/ml)
5.0000	NG
2.5000	NG
1.2500	NG
0.6250	NG
0.3125	NG
0.1563	TFTC
0.0781^b^	TFTC
0.0391^a^	(60.0 ± 9.0) × 10^1^
0.0195	(70.0 ± 5.0) × 10^2^
0.0098	(280 ± 13) × 10^3^
0.0049	(890 ± 14) × 10^5^
0.0024	TMTC
0.0012	TMTC

No growth (NG), too few to be counted (TFTC), too many to be counted (TMTC), MIC > 0.0391 µg/ml(^a^), MBC > 0.0781 µg/ml(^b^).

### Bacterial viability

**Fig. 3:**
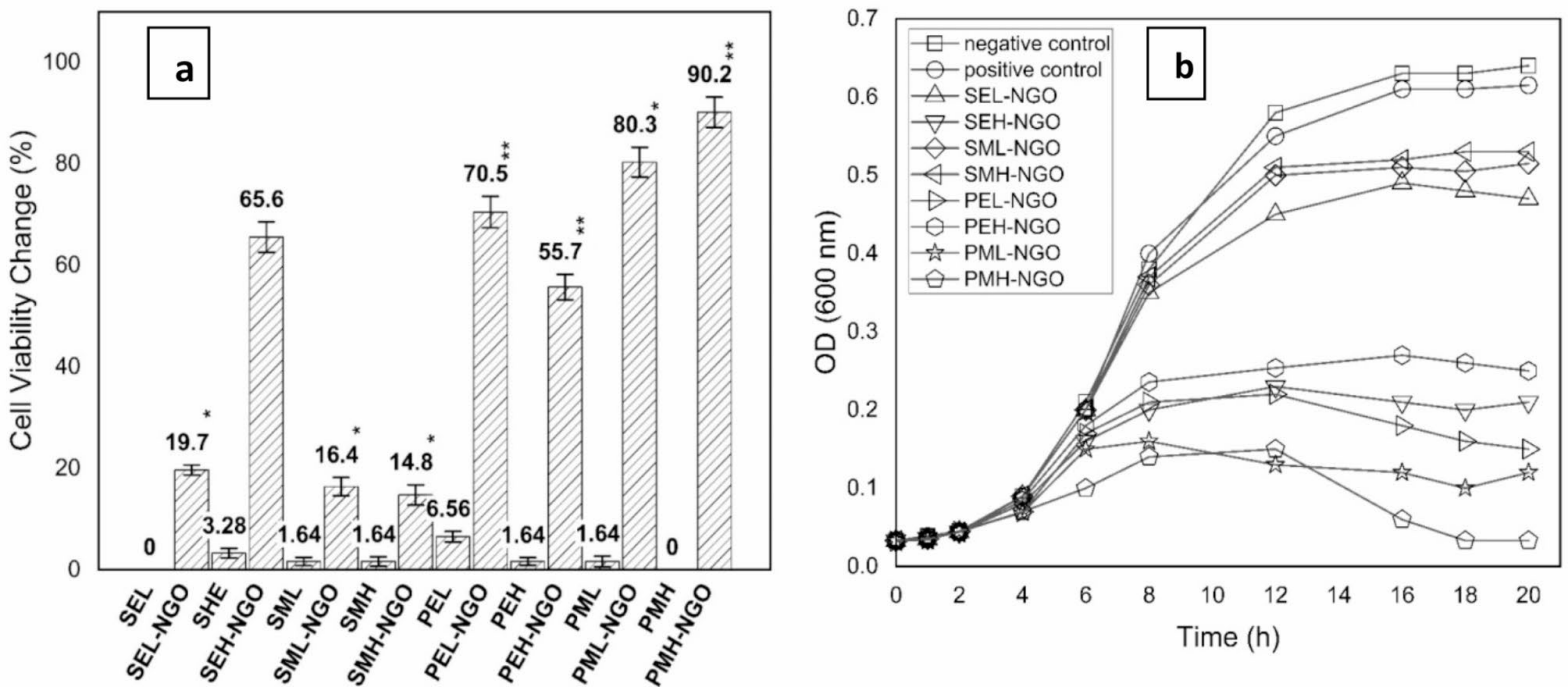
(**a**) The viability change percentages of *P. aeruginosa* samples relative to negative control bacterial count; (**b**) the time-dependent growing characteristic curves for *P. aeruginosa* samples exposed to electric and magnetic fields for 30 min and treated by GONPs (0.00244 µg/ml).

Further, the influence of exposure to electric and magnetic fields as combined with supplement of GONPs is obtained by measuring percentages of relative viability changes. The viability analysis is maintained for samples supplemented by GONPs at concentration 0.0024 µg/ml ( < < MIC) and exposed to electric and magnetic fields whereas viability changes calculated relative to samples free of exposure as negative control. For simplifying to the reader based on the treatment conditions the treated samples are coded in groups as listed in Table 2. The calculated relative viability changes are graphed in clustered columns as shown in the histogram of Fig. 3a. The obtained relative viability changes showed highly significant increase due to the synergism of the exposure with supplement of GONPs for all assigned groups. Synergistically, the highest anti-viability effect shown for samples of group PMH-GONPs by more than 90% relative change as compared to negative control followed in the order of PML-GONPs > PEL-GONPs > SEH-GONPs > PEH-GONPs. In accordance to the former findings, one may notice that the exposed samples to electric and magnetic exposure alone have not significant relative viability changes as compared with samples exposed and treated with GONPs^61^. Therefore, all further experimental analyses are done for exposed samples and treated by GONPs compared to negative control ones to investigate the optimum treatment conditions and utilize the most effective exposure parameters.

**Table 2:** List for samples grouping codes based on the treatment conditions.

Group code	Treatment condition
SEL	Static low EF (2 V/m-30 min)
SEL-GONPs	Static low EF (2 V/m-30 min) + GONPs(0.00244 µg/ml)
SEH	Static high EF (0.4 kV/m-30 min)
SEH-GONPs	Static high EF (0.4 kV/m-30 min) + GONPs(0.00244 µg/ml)
SML	Static low MF (0.112mT-30 min)
SML-GONPs	Static low MF (0.112mT-30 min) + GONPs(0.00244 µg/ml)
SMH	Static high MF (4.474mT-30 min)
SMH-GONPs	Static high MF (4.474mT-30 min) + GONPs(0.00244 µg/ml)
PEL	Pulsed low frequency EF (0.7 Hz-5.11 kV/m-30 min)
PEL-GONPs	Pulsed low frequency EF (0.7 Hz-5.11 kV/m-30 min) + GONPs(0.00244 µg/ml)
PEH	Pulsed high frequency EF (50 Hz-5.11 kV/m-30 min)
PEH-GONPs	Pulsed high frequency EF (50 Hz-5.11 kV/m-30 min) + GONPs(0.00244 µg/ml)
PML	Pulsed low frequency MF (0.7 Hz-0.242mT-30 min)
PML-GONPs	Pulsed low frequency MF (0.7 Hz-0.242mT-30 min) + GONPS(0.00244 µg/ml)
PMH	Pulsed high frequency MF (50 Hz-0.242mT-30 min)
PMH-GONPs	Pulsed high frequency MF (50 Hz-0.242mT-30 min) + GONPS(0.00244 µg/ml)

### Growing kinetics

The growing kinetics is studied by graphing the growth curves for 30 min exposed samples treated by GONPs (0.00244 µg/ml). The samples are allowed for incubation over 20 h whereas the optical density values are monitored every one hour to check cell populations. The growth curves are graphed between optical density values vs. time of incubation as represented in Fig. 3b. In this manner, the cell counts are confirmed by taking swaps from each group after 14 h of incubation and inoculated in agar plates and allowed for 24 h incubation as shown in Fig. 3a–b. The growth curves showed remarkable inhibitory effect in the order of PMH-GONPs > PML-GONPs > PEL-GONPs > SEH-GONPs > PEH-GONPs > SEL-GONPs > SML-GONPs > SMH-GONPs. It is worthy to state that the samples of groups SEL and PMH didn’t show any relative change as compared to negative control ones.

**Fig. 4:**
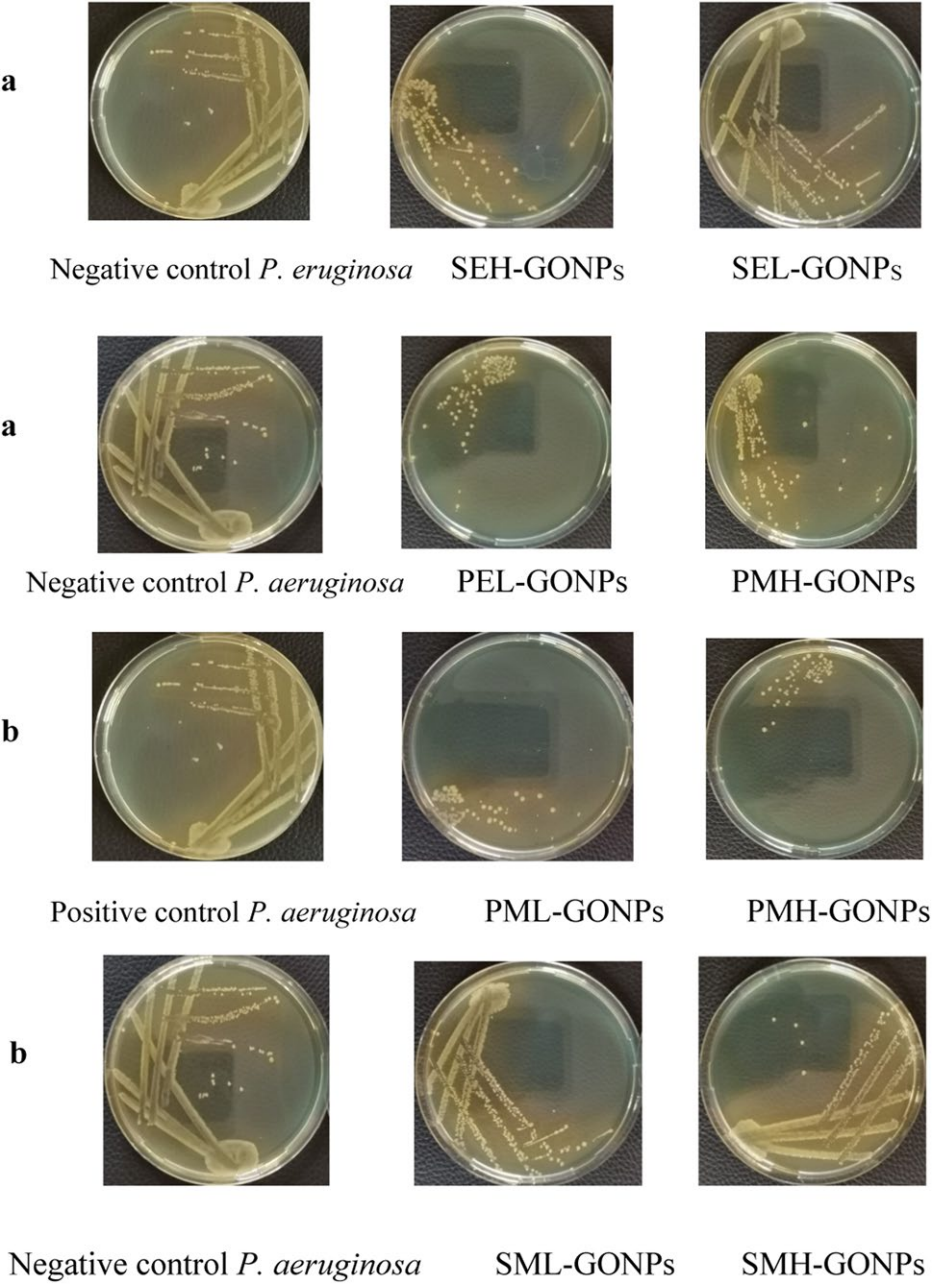
(**a**) The inoculation images for *P. aeruginosa* samples exposed to electric field for 30 min and treated by GONPS (0.00244 µg/ml) and allowed for 24 h incubation compared to positive and negative controls; (**b**) the inoculation images for *P. aeruginosa* samples exposed to magnetic field for 30 min and treated by GONPS (0.00244 µg/ml) and allowed for 24 h incubation compared to positive and negative controls.

The maximum inhibition of bacterial growth is achieved for samples of groups PML-GONPs and PMH-GONPs that are treated by GONPs and exposed to PMF-0.242mT at both assigned frequencies 0.7 Hz and 50 Hz. It is confirmed by the images of streak platted petri dishes shown in Fig. 4a. The images illuminate the few numbers of discrete countable colonies. These findings highlight the inability of exposed bacteria to grow normally as compared to negative and/or positive samples. Hence, the exposure to PMF showed potential influence against bacterial vitality in the way that it has a lethal antibacterial effect. On the other hand, potential of exposure to SEF-0.4 kV/m and PEF-0.7 Hz showed remarkable influence against bacterial growth, with almost 70% inhibition of bacterial count, as shown in Fig. 3a. The results of growing kinetics significantly confirmed the synergism between treatment by GONPS and exposure to electric and magnetic fields in such a manner that one can say that exposure is considered a co-stressor factor against bacterial growth. Furthermore, the dynamics of growth based on the exposure conditions and supplementation of GONPS are studied by calculating the arbitrary rate constant at different concentrations of GONPs^62^. The obtained arbitrary values are graphed versus GONPs concentrations as shown in Fig. 4b, whereas the curves are trended and exponential rate constants are determined as presented in Table 3. Quantitatively, the decay in the growth of bacteria is described by the negatively exponential rate constant value. As high as it is, the bacteria decay fast and are potentially influenced by the exposure parameter. The obtained data indicated that the bacterial decay is dramatically affected by the exposure to PMF-0.242mT-50 Hz which resulted in a very high exponential decay rate 251.6(µg/ml)^−1^. It is worthy to state that, for all assigned groups the potential influence of the exposure has a great antibacterial impact against bacteria as combined with GONPs in a concentration dependent manner.

### Bacteria cytotoxicity

**Fig. 5:**
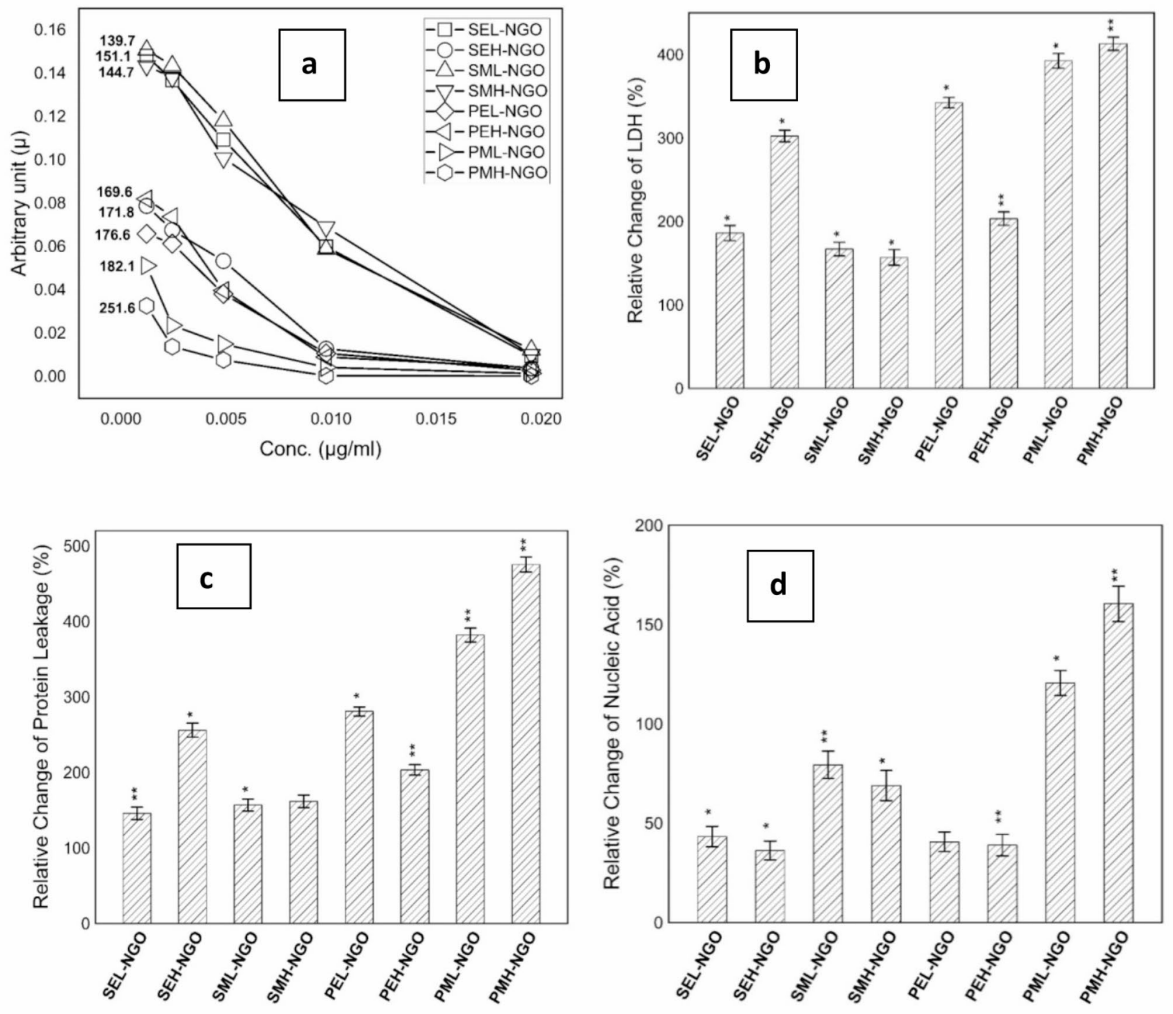
(**a**) Exponential decay curves of growth arbitrary rate constant values for *P. aeruginosa* exposed samples at different concentrations of GONPs; (**b**) relative change percentage in LDH level for exposed *P. aeruginosa* samples and treated by GONPs (0.00244 µg/ml); (**c**) relative change percentage in protein leakage for exposed *P. aeruginosa* samples and treated by GONPs (0.00244 µg/ml); (**d**) relative change percentage in nucleic acid for exposed *P. aeruginosa* samples and treated by GONPs (0.00244 µg/ml).

The direct impact of exposure on bacterial structure is determined through bacterial cytotoxicity by measuring the levels of LDH, protein leakage, and nucleic acid in exposed samples and comparing them relatively to negative control ones. The levels of LDH and protein in the extracellular medium are quantified relative to negative control samples and represented in percentages as shown in Fig. 5a–d. By comparing the levels of LDH and protein leakage for all supernatants it shows resemblance to the former growing kinetics data, the change profiles have maximum percentages for exposed samples to PMF^63^. On the other hand, the levels of nucleic acid liberated from the inside of bacterial cells into outside are obtained percentage relative to negative control samples as shown in Fig. 5d. Unlike the former results, the levels of nucleic acid indicated the highest potential of magnetic fields to penetrate inside bacterial cells to cause remarkable damage as presented by more than 70% change than control samples.

**Table 3:** List of determined exponential decay constants of trended growth arbitrary values for *P. aeruginosa* under each treatment condition at different GONPs concentrations.

Group code	SEH-GONPs	SEL-GONPs	PEL-GONPs	PEH-GONPs	PML-GONPs	PMH-GONPs	SML-GONPs	SMH-GONPs
Exponential decay constant (µg/ml)^−1^	171.8	151.1	176.6	169.6	182.1	251.6	139.7	144.7

Critically, one of the most expanding problems to the public health and human wellness is the increase of antibiotic resistant bacteria. Not as needed, the traditional methods of using antibiotics and enhance new microbial weapons are moving forward in a rate so far slower than required. To that reason the need of new nontraditional antibacterial methods became a must. Here, the hybrid of using potential of nanomaterial and exposure facilities is maintained as novel treatment against *P. aeruginosa.* The synergism of using GONPs and exposure to electric and/or magnetic fields is obtained by investigating growth characteristics and bacterial cytotoxicity. The bacteriostatic and bactericidal effects of GONPS is determined for *P. aeruginosa* samples treated with serial dilutions of GONPs (5.000 µg/ml: 0.0012 µg/ml) as tabulated in Table 1. The obtained MIC and MBC values are in agreement with recent published articles and found to be > 0.039 µg/ml and > 0.078 µg/ml respectively. The interesting surface properties of GONPS due to its functionalized oxygen group make it more electrically active relative to graphene. Therefore, its chemical composition makes it highly potent against bacteria as a result of reactive oxygen species (ROS) formation^64^. On the other hand, the compulsive physical structure of GONPS improves its antibacterial efficacy due to its sharp edges as shown in Fig. 2e. Lethally, both the functionality of oxygen and potential of sharp edges play a major role against bacteria membrane and lowering its antibacterial resistance. Furthermore, the influence of the electric and magnetic fields is determined by measuring the direct impact of the exposure synergistically with supplement of GONPs at concentration 0.0024 µg/ml ( < < MIC). It is worthy to state that the chosen concentration is too less than the MIC level to justify our hypothesis of using the exposure to electric and magnetic fields as a synergistic cofactor. The cell energy, strength and capability of surviving under the application of electric and magnetic fields are obtained as percentage of viability changes relative to the unexposed ones. The obtained viability of samples supplemented with GONPS and exposed to electric field showed significant relative changes for groups in the order of PEL-GONPs > SEH-GONPs > PEH-GONPs as shown in the histogram presented in Fig. 5d. Of note that, the electric field influences the charge distribution as it affects the movement of charged ions across bacteria cell membrane. The redistribution of the charges across the membrane alters its permeability as a result of the miss balance of the ions and molecules across it. Indeed, it modulates the ion channel and cause disruption of the ion flux and cellular process^65^. In consequence to that the cell loses its normal functionality and metabolic activity and hence its vitality as determined for samples of group PEL-GONPs in comparison to untreated ones. Remarkably, the effect of the exposure to magnetic fields didn’t show less effect than the electric one but it indicated more relative viability changes by 90% and 80% for groups PMH-GONPs and PML-GONPs respectively^66^. The obtained results illuminated the higher influence of pulsed than static magnetic fields and potential of low and high frequencies as co-stressors as combined with GONPs. Similarly, the magnetic fields have ability to reorient and direct the charges across the membrane and induce conformational changes in membrane proteins^66^. The resultant essence of these conformational changes is disrupting of essential cellular functions due to the disability of the proteins to interact with impeded molecules and/or to transport substances across the membrane^67^. Physiologically the charged residues of proteins located all over the membrane play an essential role of *P. aeruginosa* antibiotic resistance and its virulence^54–56^. The structure and function stability of the bacteria cells is intricately associated to the charges locations within micro and macro proteins allocated in the cell envelope. The negatively charged porins in the outer membrane majorly will be affected by the field interaction and thereby lose osmotic balance with irregular promotion of protein-protein interaction, substrate binding and forming of stable complexes. The confirmation of *P. aeruginosa* membrane integrity could be done by measuring the cytotoxicity of the bacteria cells in the form of the LDH levels and protein amounts leakage from inside into the outside extracellular medium^68–70^. Relative to the negative control sample the LDH and protein leakage levels showed obvious confirmation to the viability results in a way that the pulsed magnetic fields have highest synergistic antibacterial potential at low and high frequencies as presented in Fig. 5. Also, the cellular damage of exposed bacteria cells to electric fields showed significant relative changes in a way it confirms the former results. It may be presumed that the exposure had ability to alter transmembrane electrostatic potential in a manner that it allowed more GONPS to be attached to cellular surface and/or enter inside *P. aeruginosa* cells. On the other hand, the extrusion of cytoplasmic contents and cellular material as a biomarker of intercellular damage could be measured by the amount of nucleic acid leakage into outside cell in relative to the untreated samples Surprisingly, the nucleic acid leakage levels showed remarkable relative change only for samples exposed to pulsed magnetic fields namely groups PMH-GONPs and PML-GONPs with unremarkable relative changes for the other samples. Taken together, the ability of magnetic field to penetrate inside the cell and to interact directly with inter-constituents of the cell may cause the DNA destruction in synergism with GONPs of sharper edges to be inside leaded to the more of nucleic acid extrusion. Wrapping the cytotoxicity results, it confirms the bacterial fragmentation and loses of membrane integration that leaded to its dysfunctional and allowed GONPs to enter inside the cell and bonded to DNA and disrupted its replication, which in turns leaded to bacteria cell death^71,72^.

In summary, the unique structure and surface chemistry of GONPs gives ability of EMWs to enhance its interaction with bacterial cells. It may increase the oxidative stress within bacterial membrane and cause more production of ROS. The higher level of ROS leads to damaging of bacterial cellular structure. On the other hand, the influence of EMWs on charges distribution and dipole moments of bacterial membrane macromolecules results higher electrostatic interaction with negatively charged GONPs. Non-thermally, the electromechanical stress of EMWs against bacterial cell membrane leading it to be more susceptible to GONPs and increase its uptake which in turns leading to disrupt intracellular processes and cause cell death. Taking the advantage of using localized non-thermal PMF (0.242mT) at frequency range less 300 Hz in combination with GONPs as antibacterial agents, it helps in the elimination of damaging normal cells as compared to traditional treatment methods. The use of traditional methods such as plasma treatment, high voltage pulsed electric fields, and UV light exposure lead to both oxidative stress and direct physical damage to normal and bacterial cellular membrane and DNA. High voltage PEF creates electroporation, causing immediate membrane disruption and leakage of cellular contents, while UV light is highly effective at directly damaging DNA, preventing cellular replication.

To that end, our findings have several promising clinical implications in treating biofilm-associated infections on medical devices like catheters and prosthetics. The direct influence of PMF-induced GONPs delivery enhances localized treatment and antibacterial agent uptake in a way that improves treatment outcomes in chronic infections. In addition, it helps reduction of infection rates in orthopedic implants and pacemakers by preventing bacterial colonization. Moreover, one may get the benefit of using this technique to accelerate wound closure and promoting tissue regeneration. Furtherly, enhance of antibacterial agent uptake improving the chemotherapy and hence it reduces infection risks and improve therapeutic outcomes.

While our findings are promising, further extensive work to overcome some technical hurdles should be taken into account, such as the loss of nanoparticle stability, biofilm formation, and antibacterial resistance by mean time in real-world infection environments. Also, interdisciplinary collaborative work between bioengineers and clinicians should be performed to aid in controlling exposure to EMWs and ensure specific targeting in antibacterial therapies. To ensure the demonstration of the therapy safely, preclinical trials should be established, and potential environmental and health risks should be encountered.

## Conclusions

This work highlights the potential for cutting-edge applications by showcasing the distinct structural and optical characteristics of glucose-derived GONPs. Important functional groups that contribute to GONPs chemical flexibility were found by the analysis, including hydroxyl groups, ester bonds, and ether linkages. Different absorption and emission behaviors, impacted by electron-hole recombination and quantum confinement phenomena, were identified by optical characterization. With particles smaller than 35 nm, high-resolution imaging verified the nanoscale size, shape, and crystal structure. The new aspect of this work is the use of glucose as a precursor for GONPs synthesis, highlighting its versatile potential in applications needing improved optical properties and nanoscale accuracy. Our antibacterial findings confirmed the hypothesis of using the exposure to electric and magnetic fields as a synergistic cofactor in combination with GONPs. The exposure to pulsed magnetic fields showed maximum antibacterial potential as compared to the other fields. Lethally, the cytotoxicity results confirmed the ability of magnetic fields to cause bacterial fragmentation and dysfunctionality.

Magnetic field shows the ability to penetrate inside the cell and to interact directly with inter-constituents and cause the DNA destruction. Synergistically, GONPs of sharper edges leaded to the more of nucleic acid extrusion and bacteria cell death. Taken together, nanotechnology and EMWs opens a new era than traditional treatments to overcome challenges like bacterial biofilms to treat chronic or hard-to-reach infections. It holds great promise for developing effective infection control and antibacterial solutions like antimicrobial coatings for medical devices, wound healing, and even antibiotic resistance.

### Future work and scalability

On the basis of our results, it is suggested in the future work to explore different types of nanoparticles, including silver and copper, to indicate whether it is possible to generalize our synergistic hypothesis or not. The physical exposure parameters and multiple exposure techniques should be studied in the future to emphasize the optimum exposure conditions (type, frequency, time, intensity, etc.) with varying antibacterial agents (antibiotics and other nanoparticles). Future work should include different bacterial strains and multiple resistant bacteria to maximize the benefit of our hypothesized technique. Furthermore, in vivo animal model studies should explore the efficiency of our treatment hypothesis to give insights about the bioavailability, toxicity, and therapeutic potential. On the purpose of the scalability and widespread use of GONPs and EMWs, we should encounter the cost-effectiveness of nano-synthesis methods and their integration with existing medical devices. Furthermore, the development of portable devices with precise control over the different exposure parameters makes it affordable for treating localized infections. Essential comprehensive analysis of clinical trials to assess’ patient responses to comply with health regulations is a must, especially in immunocompromised populations.

## Data Availability

The datasets used and/or analyzed during the current study are available from the corresponding author on reasonable request.
